# Immediate Postoperative COVID-19 Infection after Lung Transplantation: A Systematic Review and Case Series

**DOI:** 10.3390/jcm12227028

**Published:** 2023-11-10

**Authors:** Jack K. Donohue, Eric J. Hyzny, Sarah Clifford, Ernest G. Chan, Jenalee Nicole Coster, Masashi Furukawa, Pablo G. Sanchez

**Affiliations:** Division of Thoracic Surgery, Department of Cardiothoracic Surgery, University of Pittsburgh Medical Center, 200 Lothrop Street Suite C-900, Pittsburgh, PA 15213, USA; jod154@pitt.edu (J.K.D.); cliffords2@upmc.edu (S.C.); chane@upmc.edu (E.G.C.); costerjn@upmc.edu (J.N.C.);

**Keywords:** COVID-19, lung transplantation, immunosuppression, coronavirus

## Abstract

Background: With new variants challenging the effectiveness of preventive measures, we are beginning to recognize the reality that COVID-19 will continue to pose an endemic threat. The manifestations of COVID-19 in lung transplant recipients during index admission are poorly understood with very few cases reported in recent lung transplant recipients. Optimal management of immunosuppression and antiviral therapy in recent transplant recipients is challenging. Methods: We performed a retrospective analysis identifying lung transplant recipients at our institution who contracted COVID-19 in the immediate postoperative period (within index admission). In addition, we performed a systematic review from January 2020 to August 2023 identifying all publications on the PUBMED database regarding COVID-19 infection in lung transplant recipients during index admission. Results: We report four cases of COVID-19 pneumonia in lung transplant recipients in the immediate postoperative period and we describe the clinical course, treatment options, and immunosuppression changes to manage this unique clinical problem. All patients made a full recovery and were eventually discharged home. Within our review of the literature, the most prevalent presenting symptoms were cough, dyspnea, and fatigue. Six (75%) patients decreased or held their antimetabolite. The two most common treatments were monoclonal antibodies (38%) and remdesivir (63%). Conclusion: Although previous literature demonstrates that COVID-19 can be deadly in recent lung transplant recipients, rapid treatment with anti-viral therapy/immunotherapy, deescalating immunosuppression, and treatment of respiratory decompensation with Decadron was effective in our patients.

## 1. Introduction

COVID-19 caused by the novel SARS-CoV-2 virus has a wide clinical spectrum, ranging from asymptomatic infection and mild upper respiratory tract illness to acute respiratory distress syndrome and multisystem organ dysfunction. Lung transplant recipients are at increased risk for infection and progression to fulminant disease given their frequent comorbidities and high levels of immunosuppression. The immediate postoperative period is of particular concern due to targeted T-cell induction immunotherapy. Initial reports of COVID-19 in patients with a remote history of lung transplantation show very poor outcomes [1,2,3]. Despite advances in management and antiviral therapy, optimal management of immunosuppression in lung transplant recipients with COVID-19 during index admission remains challenging with few reports available [1,2,3,4,5,6]. Here, we report four cases of COVID-19 in recent lung transplant recipients, highlighting our clinical approach to immunosuppression and anti-viral therapy over the course of the pandemic in addition to conducting a review of the existing literature.

## 2. Materials and Methods

We performed a retrospective analysis of all adult patients undergoing primary lung transplantation with immediate postoperative COVID-19 infection at our institution from January 2020 to August 2023. Patients who underwent concomitant cardiac procedure, solid organ transplantation, or lung retransplantation were excluded from this study. Immediate postoperative COVID-19 infection was defined as having a positive test during index admission.

**Figure 1:**
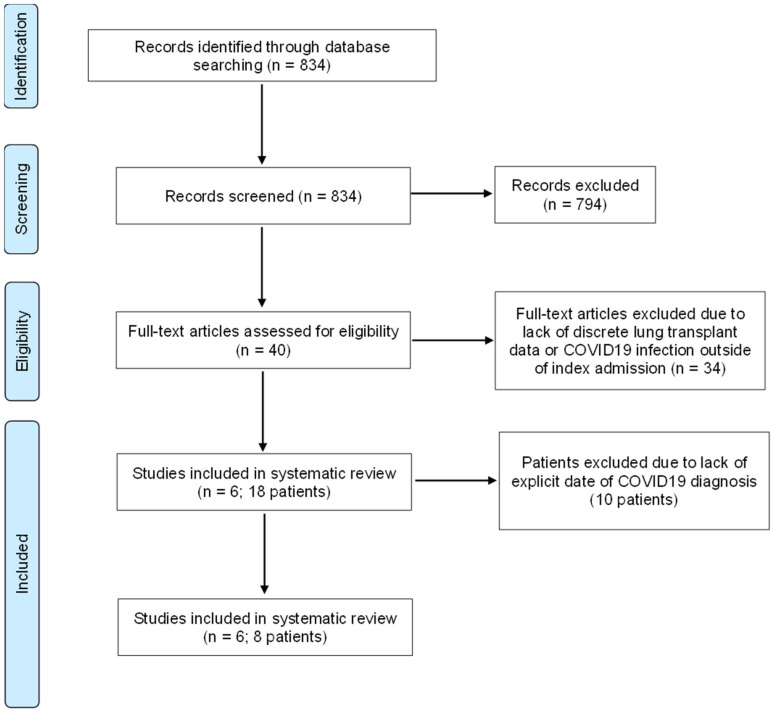
Review of the literature PRISMA flow diagram.

A systematic review was performed using PubMed database from January 2020 to August 2023. This systematic review followed guidelines established by the Preferred Reporting Items for Systematic Reviews and Meta-analyses (PRISMA). The search was restricted to publications in English. The following search terms were applied: ((lung transplantation) AND (COVID-19)), ((lung transplantation) and (coronavirus)), ((lung transplantation) and (SARS-CoV-2)). In addition, references from original papers were manually searched for relevant citations. We only included papers that contained patients with COVID-19 infection during their index admission in the postoperative setting. Publications regarding preoperative COVID-19 infection and postoperative outside of index admission were excluded from the analysis. A PRISMA flow diagram is included in Figure 1. All data were extracted from article text, tables, and figures by two independent reviewers. Discrepancies were adjudicated by the corresponding authors to retrieve data relevant for the current study.

Primary outcomes were mortality and changes in immunosuppression. The data collected also included the number of patients included, average age, patient(s) sex, transplant indication, time to diagnosis, comorbidities, presenting symptoms, COVID-19 diagnostic methods, imaging modality, imaging findings, hospital length of stay, antibiotic/antiretroviral treatment, and O_2_ support method. All studies were available for synthesis and variables of interest that were not reported, were marked as “NR” for the respective study. A meta-analysis could not be performed due to the heterogeneity of literature collected and the limited quantity of literature in this area. The protocol for this systematic review was not registered.

A pre-planned risk of bias was not assessed within this systematic review due to the lack of high-quality evidence investigating immediate postoperative COVID-19 infection in lung transplant recipients. The systematic review consists of case reports and case series studies that contain inherent biases and the lack of a control group.

## 3. Results

### 3.1. Case Series

**Figure 2:**
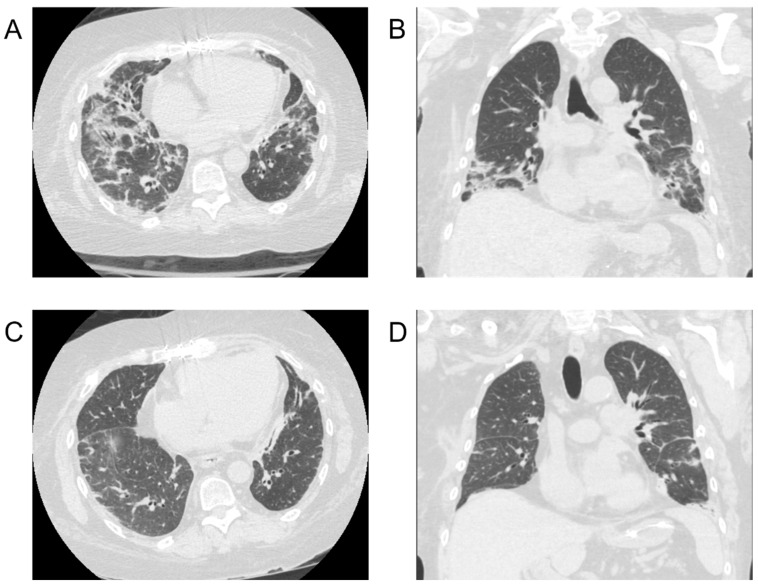
Case 4. CT scans of SARS-CoV-2 infection. (**A**) Axial image 18 days after diagnosis; bilaterally moderate patchy ground glass opacities in the lower lobes. (**B**) Coronal image 18 days after diagnosis; bilaterally moderate patchy ground glass opacities in the lower lobes. (**C**) Axial image 52 days after diagnosis demonstrating exceptional recovery of lung parenchyma. (**D**) Coronal image 52 days after diagnosis demonstrating exceptional recovery of lung parenchyma.

Four patients tested positive for COVID-19 within their index hospitalization after primary lung transplantation. Three tested positive in early 2021 and one in early 2022. All four were transplanted for restrictive lung disease. The average LAS was 40 and the average age was 62 years. Three were diagnosed by routine nasal swab prior to discharge to acute rehab with no other symptoms, while one was tested for new mild shortness of breath and cough. Computed tomography (CT) in two patients showed multi-lobar opacities concerning for multi-lobar pneumonia. Two patients showed classical ground glass opacities on imaging (Figure 2). Case 1 presented with severe COVID disease and required 40 L high flow nasal canula and was transferred to the ICU. He was treated with Remdesivir in addition to a course of steroids. Patient 2 was the only to have been previously vaccinated for COVID-19 (mRNA) having received three doses with the last dose 5 months prior to lung transplantation. He developed moderate pneumonia that required 3 L nasal canula for several days. He was treated with monoclonal antibodies and high dose steroids. The remaining two patients had mild disease and required no oxygen. One of these patients (case 3) received Remdesivir in addition to convalescent plasma, while the remaining patient only received monoclonal antibodies. Two patients (cases 1 and 3) were treated with empiric broad spectrum antibiotics for presumed bacterial superinfection. Three patients were anticoagulated for history of stroke, factor V Leiden and perioperative atrial fibrillation. The remaining patient was anticoagulated with prophylactic heparin. Three patients received Simulect induction due to their age or comorbidities. Mycophenolate was held in all patients, initiated at half dose just prior to discharge and increased to full dose 2 weeks later. All patients made a full recovery and were eventually discharged home. All four were still testing positive on discharge. The average length of positivity was 92.5 days. The biopsies at 6 months and 1 year post-transplant showed no evidence of acute rejection. PFTs at 6 months showed diminished FEV1 and FVC at an average of 68% and 60%, respectively.

### 3.2. Systematic Review

A total of 40 records were identified and eligible for full-text review. After reviewing for eligibility, we found two case series and four case reports of patients with COVID-19 infection in the immediate post-operative period (within index admission) after lung transplantation, totaling 18 patients [1,2,3,4,5,6]. However, 10 of these patients did not have explicitly reported COVID-19 diagnosis dates, were infected outside of the immediate post-operative period, or had insufficient data reported and were excluded from this analysis. All literature included in the current systematic review was published from 2020 to 2022. This literature is summarized in Table 1. A manual search of references did not identify any additional studies.

Of lung transplantation patients with immediate post-operative COVID-19 and available data, the mean age at the time of COVID-19 diagnosis was 59 years (range: 37–71 years) and 50% were male. Reasons for transplantation in the cohort with immediate post-operative COVID-19 were as follows: idiopathic pulmonary fibrosis (50%), hypersensitivity pneumonitis (33%), and cystic fibrosis (17%). The mean interval between the date of transplant and the date of COVID-19 diagnosis was 12 days (range: 1–22 days). All patients were under immunosuppressive therapy for rejection prophylaxis at time of COVID-19 diagnosis.

The most prevalent presenting symptoms were as follows: dyspnea (80%), cough (60%), and fatigue (60%). GI symptoms were less common at 20%. All patients had RT-PCR tests run on samples collected via nasopharyngeal swab with fewer having additional samples collected via oropharyngeal swab (33%), saliva (17%), and bronchioalveolar lavage (17%) (n = 2, 6.1%). All patients diagnosed in the immediate postoperative period tested positive via RT-PCR. The majority of patients underwent CT scans (88%) and to a lesser degree chest X-ray (25%). The most common findings on radiographical imaging were ground glass opacities (75%) and consolidation (38%). Chest X-ray was clear in one (13%) patient.

The management of COVID-19 infections in lung transplantation recipients is summarized in Table 2. In terms of alterations in immunosuppressive regimen management, six (75%) patients decreased or held their use of antimetabolites, two (25%) decreased or held their use of calcineurin inhibitors, and two (25%) decreased or held their use of steroids. The two most common antibiotic/antiviral treatments were monoclonal antibodies (38%) and remdesivir (63%). However, there were patients that also received hydroxychloroquine (13%), zosyn (13%), azithromycin (13%), meropenem (13%), and no antibiotic/antiviral treatment (13%). None of the patients received oseltamivir for the treatment of their immediate postoperative COVID-19 infection. Mechanical ventilation was required in 63% of patients with one (13%) requiring mechanical ventilation in the prone position. None of the patients with immediate COVID-19 infection after lung transplantation required extracorporeal membrane oxygenation.

The mean hospital length of stay for patients with available data was 52 days (range: 14–122 days). Of the eight cases of COVID-19 infection in the immediate post-operative period after lung transplantation, three (37.5%) succumbed to infection.

## 4. Discussion

Here, we report four cases of lung transplant recipients diagnosed with COVID-19 during their initial hospitalization an average of 26 days post-transplant, despite both donor and recipient testing negative at time of the initial operation. The initial diagnosis of COVID-19 in recent lung transplant recipients is challenging. Classical signs and symptoms may be masked due to the recency of the transplant operation, initiation of new medications, primary graft dysfunction, or other infectious etiologies. Three of the four patients displayed no symptoms at the time of diagnosis and the remaining patient reported mild shortness of breath and worsening cough. No patients developed objective fever over the course of the acute infection. However, in the reported literature, fever is not a common presentation in lung transplant recipients who contract COVID-19 [2,3,4,5]. Despite investigation and contact tracing, no source of COVID-19 was identified. Patient support persons and hospital staff with direct patient contact were tested in accordance with hospital protocol and all returned negative. It is important to note that three of the four patients were not vaccinated at time of transplant as vaccines were not yet widely available. The only patient who required high-flow supplemental oxygen was vaccinated. Due to the ever-changing genotypes of COVID-19 and what vaccines are currently effective for, we continued to see COVID-19 after lung transplant independent of vaccination status.

Without consensus guidelines to define treatment algorithms in this cohort, treatment decisions were made by a multidisciplinary COVID team, in conjunction with transplant surgery. Treatment strategies with antiviral and neutralizing antibody modalities continue to rapidly evolve with no clear gold-standard treatment. Two of our patients received Bamlanivimab as neutralizing antibody monotherapy, while the other two patients received a 5-day course of Remdesivir. This was in accordance with the literature review demonstrating that monoclonal antibody and Remdesivir were the two most common treatments. Patient treatment selection depends upon the COVID-19 disease severity and Remdesivir is indicated in patients with severe disease while Bamlanivimab is recommended for patients with mild to moderate disease severity at risk of progression [7]. Both Bamlanivimab and Remdesivir have been shown to reduce mortality in hospitalized patients with COVID-19. More recent evidence supports the combination of Bamlanivimab with Etesevimab, yielding decreased viral load when compared to placebo or Bamlavinimab alone [8,9]. Anti-viral therapy was patient specific, based on ongoing trials and the best available evidence at the time. At present, immunocompromised patients with COVID-19 pneumonia without an increase in oxygen requirement receive monoclonal antibody treatment. Patients requiring greater than 6 L of supplemental oxygen are treated with a 5-day course of Remdesivir and 6 mg dexamethasone daily for 10 days or until they no longer require oxygen. Since recent transplant patients merit more aggressive treatments, our first patient was treated with a course of Remdesivir despite not requiring oxygen. Any increase in oxygen requirement after diagnosis of COVID-19 in our lung transplant recipients was treated with 6 mg dexamethasone daily for 10 days. Multiple randomized controlled trials have demonstrated the survival benefit of steroid therapy for COVID pneumonia [10,11] and it is the practice of our group to treat recent transplant recipients liberally as soon as there is an increase in oxygen requirement. Two patients had an increase in oxygen requirement, and both received dexamethasone in accordance with the above protocol. All patients fully recovered from the initial infection with an average follow-up of 469 days. Pulmonary function testing shows mildly diminished function with three of the four patients demonstrating restrictive pattern of lung disease. There was no evidence of post-acute sequela of SARS-CoV2 infection or “long-COVID”.

Balancing immunosuppression against the risk of infection is a challenging clinical problem under normal circumstances. Infection with SARS-CoV-2 complicates the matter further. In the transplant literature, pausing antimetabolite therapy, but continuing calcineurin inhibitors is a common management strategy [1,2,4,5,12]. Mycophenolate has been suggested to increase the risk of severe COVID-19 in a dose dependent manner in liver transplant recipients [13]. Antimetabolite therapy inhibits the proliferation of lymphocytes, and its discontinuation is believed to bolster cell-specific immunity [14]. However, it has been suggested that patient outcomes in recent liver transplant recipients with COVID-19 are due more to patient comorbidities than immunosuppression [15]. We chose to hold mycophenolate therapy in all four of our recent transplant recipients with COVID-19. Despite continued positive tests, all patients had clinically recovered prior to discharge, and mycophenolate was resumed at half dose 1 day prior to discharge with increase to full therapeutic dose 2 weeks later. It remains our practice to hold antimetabolite therapy but continue calcineurin inhibitors during the acute infection. This was in accordance with the literature review demonstrating six (75%) patients decreased or held their antimetabolite and two (25%) decreased or held calcineurin inhibitor. With the brief pause of immunosuppression, no patient developed any events of acute rejection at 6 months or 1 year.

In the current review of eight lung transplant recipients with COVID-19 infection in the immediate postoperative period, the mortality rate was 37.5%, which is disparate from our case series where all four patients survived to discharge. The mortality results of our literature review are in general agreement with the current COVID-19 literature as this viral infection has been found to be associated with increased risk of both postoperative complications and 30-day mortality in patients who contract the COVID-19 virus in an immediate postoperative setting [16,17,18]. During the critical time period of recovery from lung transplantation, it is therefore vital to ensure these patients are not exposed to the virus. At our institution, all staff entering or examining lung transplant recipients independent of the time from surgery are mandated to wear personal protective equipment (PPE) at each encounter and to practice hand hygiene. In addition, family support in the postoperative period is restricted to one individual who is also required to wear PPE at the time of interaction with the patient.

All four of our patients were stable for discharge despite continuing to test positive for COVID-19. Moderately or severely immunocompromised individuals may produce replication-competent virus beyond 20 days [19]. This presents a significant challenge in recent transplant recipients, who require high levels of contact with health care workers and home health aid. In our series, patients continued to test positive through RT-PCR of respiratory samples an average of 95 days after initial positive test, despite being symptom free. These findings are in line with two case reports that reported duration of positive tests [4,5]. It is important to recognize that severely immunocompromised patients with COVID-19 are capable of viral spread if respiratory samples remain positive and proper respiratory precautions are imperative during all patient interactions. Patients are considered at risk of transmission until they receive two negative tests greater than 24 h apart. Three of the four patients received their primary vaccination series while still testing positive. All four patients had received two vaccine series and two booster doses. It has been shown that vaccine efficacy and antibody response to infection is reduced in immunocompromised individuals [18]. With continued vigilance and respiratory precautions, none of the four patients contracted COVID-19 after their initial infection. They remain in good health.

There are several limitations associated with our case series and literature review findings. First, due to the rarity of the current topic, the outcomes reported in the current study are based on a small group of patients and may lack power. Due to the small sample size, the effects of variations in lung transplant practices as well as treatment strategies across centers can confound the results. Lastly, because of the short follow-up associated with the studies included, we can only report on immediate results and cannot offer any insight into important clinical questions such as the long-term effects of the COVID-19 virus on graft survival in lung transplant recipients. Despite these limitations, we include the most comprehensive group of lung transplant recipients who have contracted the COVID-19 virus during their index admission to date. To increase statistical power and identify risk factors associated with poor outcomes, a large multicenter investigation into the impact of COVID-19 on lung transplant recipients in the immediate postoperative period is warranted. In addition, investigating the long-term overall survival, graft survival, and pulmonary function in lung transplant recipients who acquire COVID-19 during the index hospital admission will inform post-discharge care and follow-up visit intervals.

## 5. Conclusions

Recommendations for the treatment of COVID-19 in lung transplant patients defer to those for the general population where treatment is based on disease severity. Here, we describe four cases of COVID-19 immediately after lung transplantation. Although COVID-19 pneumonia can be deadly in this unique cohort, rapid treatment with anti-viral therapy/immunotherapy, deescalating immunosuppression, and treatment of respiratory decompensation with Decadron was effective in our patients. Despite reduction in immunosuppression, no patient developed episodes of acute rejection.

## Figures and Tables

**Table 1:** Literature review of lung transplant recipients who contracted the COVID-19 virus in the immediate post-operative period. * Immediate postoperative infection, Afib atrial fibrillation, CAD coronary artery disease, CF cystic fibrosis, CHD coronary heart disease, CHF congestive heart failure, CKD chronic kidney disease, COPD chronic obstructive pulmonary disease, CT computer tomography, CXR chest X-ray, DM2 diabetes mellitus type II, DVT deep venous thrombosis, GERD gastro-esophageal reflux disease, GGO ground-glass opacities, HF heart failure, HL hyperlipidemia, HP hypersensitivity pneumonitis, HTN hypertension, IPAF interstitial pneumonia with autoimmune features, IPF idiopathic pulmonary fibrosis, NR not reported, OSA obstructive sleep apnea, PHTN, pulmonary hypertension, PVD peripheral vascular disease.

Author (Year)	Number of Patients	Age (Range)	Sex (nF, nM)	Transplant Indication	Time to Diagnosis (Days)	Comorbidities	Presenting Symptoms	COVID-19 Diagnostic Methods	Imaging Modality	Imaging Findings	LOS, Days	Survived Infection, n (%)
Yokoyama et al. (2022) [5]	1	71	(0, 1)	HP	1	None	Cough, Dyspnea, Fatigue	Nasopharyngeal Swab, Bronchoalveolar Lavage, Saliva	CXR	None	14	1/1 (100)
Faccioli et al. (2022) [4]	1	52	(1, 0)	IPF	12	NR	Cough	Nasopharyngeal Swab	CT	Consolidation	NR	1/1 (100)
Zimmermann et al. (2022) [1]	6	60 (38–69)	(4, 2)	IPF (3), IPAF (1), HP (1), Sarcoidosis (1)	NR (4), 18, 22	HF (3), PHTN (3), HTN (2), PVD (1), CHD (1), Afib (1)	Fatigue (6), Dyspnea (5), Cough (5), Diarrhea (4), Fever (3), Asthenia (3), Nausea (2), Expectoration (2), Headache (2), Myalgia (1), Abdominal Pain (1)	Nasopharyngeal and Oropharyngeal Swab	CT	GGO (6), Consolidation (4), Intersitial Abnormalities (4), Pleural Effusion (3)	50 (25–122)	4/4 (100), 1/2 * (50)
Myers et al. (2020) [2]	8	60.8 (43–75)	(1, 7)	IPF (5), COPD (2), CF (1)	NR (6), 7, 14	Prior Smoker (7), Afib (3), CKD (3), DM2 (3), HL (3), DVT (2), HTN (2), CHF (1), CAD (1), OSA (1)	Cough (6), Dyspnea (6), Fever (4), Nausea (3), Fatigue (3)	NR	CT	GGO, Consolidation	8.6 (2–16)	6/6 (100), 0/2 * (0)
Athanazio et al. (2020) [6]	1	37	(0, 1)	CF	13	None	Dyspnea	Nasopharyngeal Swab	CT	GGO, Consolidation, Pleural Effusion	39	1/1 (100)
Keller et al. (2020) [3]	1	69	(1, 0)	IPF	8	GERD, HL, Psoriasis	NR	Nasopharyngeal Swab	CXR, CT	GGO, Consolidation	NR	1/1 (100)

**Table 2:** Management of active COVID-19 infections in lung transplant recipients in a hospital setting. CNI calcineurin inhibitor, ECMO extracorporeal membrane oxygenation.

Changes in Immunosuppression	n (%)
Decrease or Hold Antimetabolite	6 (75)
Decrease or Hold CNI	2 (25)
Decrease or Hold Steroids	2 (25)
Antibiotic/Antiviral Treatment of Choice	
Monoclonal Antibody	3 (38)
Hydroxychloroquine	1 (13)
Remdesivir	5 (63)
Oseltamivir	0 (0)
Zosyn	1 (13)
Azithromycin	1 (13)
Meropenem	1 (13)
None	1 (13)
O_2_ Support	
Mechanical Ventilation	4 (50)
Mechanical Ventilation + Prone Positioning	1 (13)
ECMO	0 (0)

## Data Availability

The data that support the findings of this study are available from the corresponding author upon reasonable request.
